# Flip-flop footwear with a moulded foot-bed for the treatment of foot pain: a randomised controlled trial

**DOI:** 10.1186/s12891-016-1327-x

**Published:** 2016-11-11

**Authors:** Vivienne Helaine Chuter, Angela Searle, Martin J. Spink

**Affiliations:** 1 School of Health Sciences, The University of Newcastle, 10 Chittaway Road, PO Box 127, Ourimbah, NSW 2258 Australia; 2 Priority Research Centre for Physical Activity and Nutrition, The University of Newcastle, Callaghan, Australia

**Keywords:** Shoes, Pain measurement, Health status indicators

## Abstract

**Background:**

Foot pain is a common problem affecting up to 1 in 5 adults and is known to adversely affect activities of daily living and health related quality of life. Orthopaedic footwear interventions are used as a conservative treatment for foot pain, although adherence is known to be low, in part due to the perception of poor comfort and unattractiveness of the footwear. The objective of this trial was to assess the efficacy of flip-flop style footwear (*Foot Bio-Tec©)* with a moulded foot-bed in reducing foot pain compared to participant’s usual footwear.

**Methods:**

Two-arm parallel randomised controlled trial using computer generated random allocation schedule at an Australian university podiatry clinic. 108 volunteers with disabling foot pain were enrolled after responding to an advertisement and eligibility screening. Participants were randomly allocated to receive footwear education and moulded flip-flop footwear to wear as much as they were comfortable with for the next 12 weeks (*n* = 54) or footwear education and instructions to wear their normal footwear for the next 12 weeks (*n* = 54). Primary outcome was the pain domain of the Foot Health Status Questionnaire (FHSQ). Secondary outcomes were the foot function and general foot health domains of the FHSQ, a visual analogue scale (VAS) for foot pain and perceived comfort of the intervention footwear.

**Results:**

Compared to the control group, the moulded flip-flop group showed a significant improvement in the primary outcome measure of the FHSQ pain domain (adjusted mean difference 8.36 points, 95 % CI 5.58 to 13.27, *p* < 0.01). Statistical and clinically significant differences were observed for the secondary measure of foot pain assessed by a VAS and the FSHQ domains of foot function and general foot health. None of the participants reported any pain or discomfort from the intervention footwear and six (footwear group = 4) were lost to follow up.

**Conclusions:**

Our results demonstrate that flip-flop footwear with a moulded foot-bed can have a significant effect on foot pain, function and foot health and might be a valuable adjunct therapy for people with foot pain.

**Trial registration:**

ACTRN12614000933651. Retrospectively registered: 01/09/2014.

## Background

Foot pain is widely acknowledged to be a common problem. A recent systematic review involving 75,000 adults found that approximately 1 in 5 people over 45 years of age experience ‘frequent’ foot pain [1], and a UK survey of 3,417 adults reported 24 % of females and 20 % of males had foot pain in the last month lasting at least 1 day [2]. In the middle aged and older population, foot pain is more prevalent in females, the forefoot and toes are the most common site of pain, and in two thirds of cases this results in a moderate disability in an aspect of daily life [1]. The community burden of foot pain is high and is known to adversely affect activities of daily living [2], reduce health related quality of life (QOL) [3], impair balance and functional ability in community dwelling older people [4], and increase risk of falls in older people [5, 6].

Footwear interventions are widely used as a conservative treatment method for foot pain [7, 8]. The orthopaedic footwear usually recommended is a closed in lace-up style shoe and adherence to this intervention is known to be as low as 22 %, with perceived poor comfort and unattractiveness of the footwear cited as the main-reasons for non-adherence [8, 9]. Flip-flop style footwear has not been considered as a treatment modality for foot pain as it is believed to provide less support, protection, cushioning and motion control compared to closed-in shoes [10–12]. The introduction of new design features in flip flops such as moulded foot-beds, increased sole thickness and a heel-to-forefoot slope allows them to provide some of the functionality of closed in shoes [13, 14]. Despite the popularity of flip-flop style of footwear, there is very limited evidence regarding the effect these design features have on foot pain and foot function, with only one study investigating the use of a contoured sandal for treating plantar heel pain [15]. This study found the sandal provided a similar beneficial reduction in pain compared to a contoured shoe insert, and that both the sandal and the shoe insert provided a significant reduction in pain compared to a flat flip flop. The aim of this study was to investigate the efficacy of flip-flop style footwear with a moulded foot-bed in reducing foot pain and improving function compared to usual footwear in adults with disabling foot pain. We also assessed the effect of the footwear on perceptions of general foot health and comfort.

## Methods

### Design and trial registration

The study was a two-arm parallel group randomised controlled trial with a 12 week follow up period and is reported using CONSORT guidelines [16]. The trial has been registered on the Australian New Zealand Clinical Trials Registry (ACTRN12614000933651). All participating patients gave their informed consent. Research was conducted at the University of Newcastle Podiatry Clinic at Wyong Hospital.

### Randomisation and blinding

After enrolment participants completed a set of baseline questionnaires to determine their foot pain and foot function. Participants were then randomised into either an intervention group that received a pair of test footwear and footwear education or a control group that received footwear education only. Prior to recruitment AS prepared sequentially numbered, opaque sealed envelopes containing a computer generated random allocation schedule with mixed block lengths of four and six participants. The person administering the intervention (VC) enrolled participants and assigned participants to groups by selection of the next sequential envelope. Statistical analysis was performed independently by MS. All outcome data was collected using self-reported questionnaires completed by the participants. The participants and the person administering the intervention (VC) were not blinded as they were aware of what type of shoe they were wearing. The person conducting data analysis (MS) was blinded to group allocation.

### Participants

Potential participants were recruited on a volunteer basis via advertising flyers at the University of Newcastle Central Coast Campus and the University of Newcastle Podiatry Clinic at Wyong Hospital. Inclusion criteria were adults, 18 years of age and over with disabling foot pain, which was defined as current foot pain located below the level of the ankle joint which prevented them from doing at least one of their normal activities. Exclusion criteria were history of foot amputation, current foot pain due to arterial insufficiency, venous insufficiency or peripheral neuropathy of any cause, a neurodegenerative disorder or a history of two or more falls in the previous 12 months. All potential participants received a participant information statement and were screened via a short phone call to ensure inclusion and exclusion criteria were met. Any participants reporting medical history associated with the exclusion criteria relating to peripheral arterial disease, venous insufficiency or peripheral neuropathy underwent additional screening including visual examination, continuous wave Doppler and/or a four site 10 g monofilament test [17, 18]. Presence of a monophasic waveform or loss of sensation at one site was considered indicative of pathology [17, 18] and the potential participant was excluded. History of falls in the last 12 months was confirmed through verbal questioning. At their first appointment participants completed the consent form before being enrolled in the trial.

### Intervention

**Fig. 1:**
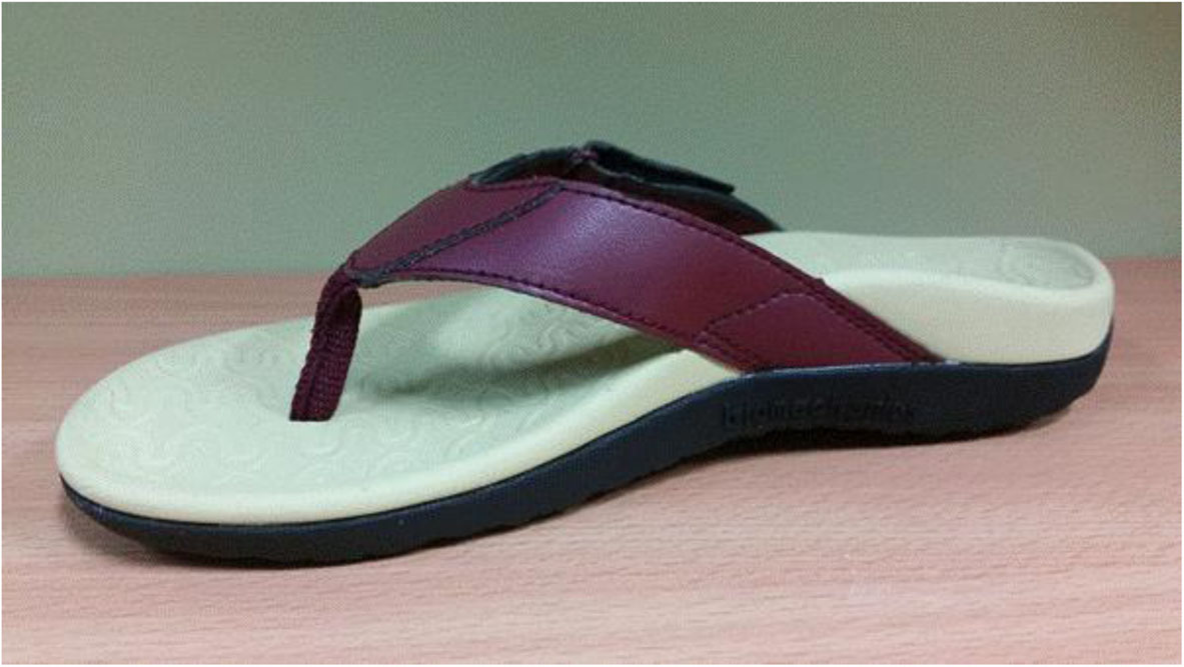
Test Footwear*: Foot Bio-Tec©* Orthotic footwear

Participants in the intervention group were fitted with a pair of flip flops (*Foot Bio-Tec©*, Silverwater, NSW, Australia) with a moulded foot-bed, heel cup and wide straps (Fig. 1), and asked to wear the footwear as much as they were comfortable with for the next 12 weeks. Participants in the control group were asked to wear their usual footwear for the next 12 weeks. Both the control and intervention groups received general advice on footwear including choosing footwear of an appropriate size and fit, with firm fastening, low heel height and slip-resistant soles. Participants who received the test footwear were asked to complete a daily footwear diary to record how often and for how long they wore the intervention footwear. At the conclusion of the trial participants were asked if they had undertaken any other treatment for their foot pain during this time and if the intervention footwear had caused any pain or discomfort.

### Outcomes

All outcome data was collected using self-reported questionnaires completed by the participants. The participants were not blinded as they were aware of what type of shoe they were wearing.

### Primary outcome measure

The primary outcome measure was the foot pain domain of the Foot Health Status Questionnaire (FHSQ) recorded at baseline and the 12 week follow-up. The FHSQ measures foot specific health related QOL and has been shown to demonstrate high content, criterion and construct validity as well as clinical utility and reliability [19, 20]. The FHSQ measures four subscales of foot health: foot pain, foot function, footwear and general foot health. Scores are converted to a scale from 0, which represents the poorest state of foot health, to 100 which signifies optimum foot health. The minimal important difference (MID) value used for this domain was 14 points [21].

### Secondary outcome measures

Secondary outcome measures, recorded at baseline and the 12 week follow-up, were the FHSQ domains of foot function and general foot health as described above, plus current foot pain using a 100 mm visual analogue scale which is widely used due to its simplicity and adaptability [22, 23] and has been validated as a reliable generic pain measure [24]. The instructions on the VAS stated ‘Please mark the position on the line that represents how much foot pain you are currently experiencing. The far left end indicates ‘No pain’ and the far right end indicates ‘Worst pain imaginable”. Foot pain intensity on the VAS is rated from no pain (score of 0) to worst imaginable pain (score of 100). Minimal important difference (MID) values used for these measures were 9 mm for pain on the VAS and with the FHSQ, 7 points for foot function and 9 points for general foot health [21].

Perceived comfort of the intervention footwear was also recorded at 12 weeks following the intervention. This was measured using a VAS based survey that collected data on the comfort level of the heel, midfoot and forefoot and the overall comfort of the footwear. The instructions on the VAS stated ‘Please mark on the lines below to indicate how you would rate your shoes for each of the components of the shoe. The further to the right you mark the line the more comfortable you find that aspect of the shoe.’ Comfort was rated from 0 mm (not comfortable at all) to 100 mm (most comfortable condition imaginable). It has been reported previously that VAS scales are reliable in assessing footwear comfort [25].

### Sample size

We based the a priori sample size calculations on a minimal important difference of 14 points for the foot pain domain of the FHSQ [21]. Assuming a standard deviation of 20.5 points based on the average standard deviation of previously reported baseline FHSQ pain measures [26], a power of 90 % and an alpha 0.05 and allowing for 20 % attrition rate, 54 participants per group were required making a total sample size of 108 participants.

### Statistical analysis

Statistical analysis was undertaken using SPSS version 21 (IBM Corp, Chicago, Illinois) by a researcher blinded to group allocation. Statistical significance was delimited at *P* < 0.05. Data were assessed for normality of distribution, internal consistency, homogeneity of variance and linearity. The difference between groups at follow-up for the primary outcome (foot pain domain of the FHSQ) was analysed with analysis of covariance (ANCOVA) using a linear regression approach. We pre-specified that the baseline measure of FHSQ pain was used as the only covariate in the analysis. Similarly for the secondary outcome measures of FHSQ function, FHSQ general foot health and the VAS for foot pain, ANCOVA using a linear regression approach was conducted. The baseline measures of FHSQ function, FHSQ general foot health and the VAS for foot pain were the only covariates used in each respective analysis. Cohen’s d was used to calculate effect sizes for the primary and secondary outcomes. An effect size of greater than or equal to 0.8 was considered to represent a large clinical effect, 0.5 a moderate effect and 0.2 a small effect [27]. Comfort ratings were calculated as a mean score for each category of the survey.

Data were analysed by intention to treat. Missing data were estimated in SPSS using multiple imputation with a regression method for all continuous variables [28]. Ten imputed data sets were generated for analysis and the results of each combined. The data contained only monotone missing patterns with six participants lost to follow up (four from the intervention group, two from the control group) representing 5.5 % of the data.

## Results

**Fig. 2:**
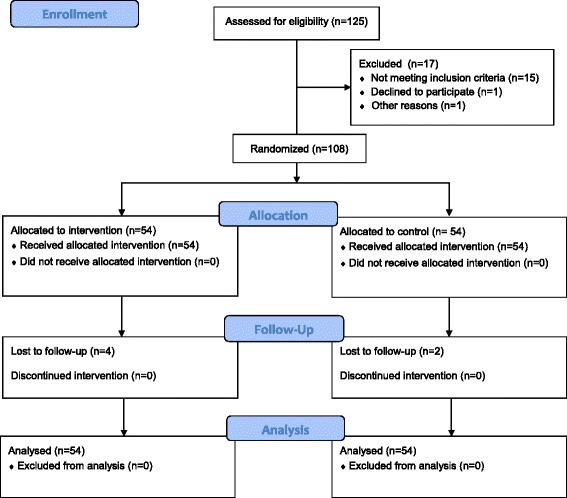
Flow of participants through the randomised controlled trial

One hundred and eight participants were recruited between April and October 2014. Participants’ progression through the trial is shown in a CONSORT diagram in Fig. 2. Participant baseline characteristics are included in Table 1. Mean hours of flip-flop use by the intervention group over the 12 week period was 183.03 h (SD 75.69 h) which equates to approximately 15.25 h per week.

**Table 1:** Participant characteristics at baseline

Characteristic	Control (*n* = 54)	Flip-Flop (*n* = 54)
Sex	Male 23 (43)	Male 27 (50)
	Female 31 (57)	Female27 (50)
Age (mean ± SD in years)	49.1 ± 16.9	48.6 ± 14.1
Duration of foot pain (mean ± SD in weeks)	71.3 ± 179.3	63.5 ± 163.4
Location of pain		
Rearfoot	11 (20)	15 (28)
Midfoot	12 (22)	11 (20)
Forefoot	22 (41)	18 (33)
General foot	9 (17)	10 (19)
Does regular exercise	49 (87)	47 (85)
Medical Conditions^a^		
Diabetes	3 (5.5)	8 (14.8)
Osteoarthritis	12 (22.2)	10 (18.5)
High blood pressure	24 (44.4)	23 (42.6)
Heart disease	4 (7.4)	5 (9.3)
Lung disease	6 (11.1)	2 (3.7)
Hormone replacement therapy	0 (0)	3 (5.5)
Hypercholesterolemia	16 (29.6)	23 (42.6)
Rheumatoid arthritis	1 (1.9)	2 (3.7)
Back pain	8 (14.8)	11 (20.4)
Depression	5 (9.3)	5 (9.3)

Values are number (%) unless otherwise stated^a^Some participants reported multiple medical conditions

None of the participants reported any pain or discomfort from wearing the intervention footwear. Fifty (94 %) participants in the intervention group submitted their footwear diary and the comfort questionnaire at the completion of the trial. Both groups reported the use of co-interventions during the trial period. The most common co-interventions were analgesic use (35 % of control group and 30 % of intervention group) and further podiatry treatment including general treatment, taping and stretching (22 % of control group and 26 % of intervention group).

### Primary outcome

When compared to the control group, participants in the flip-flop intervention group demonstrated statistically significant improvements in pain measured at 12 weeks by the foot pain domain of the FHSQ (adjusted mean difference 9.6, 95 % CI:5.5 to 13.3) with a moderate effect size (Cohen’s *d 0.64)* (Table 2). The improvement in pain for the foot pain domain of the FHSQ was less than the MID for a clinically meaningful difference in pain levels.

**Table 2:** Primary and secondary outcome measures of pain and function at baseline and 12 week follow-up. Values are means (standard deviations) unless otherwise stated

Measure	Intervention Group		Control group		Adjusted mean difference (95 % CI)	*P* Value	Effect size (Cohen’s d)
	Baseline	Follow-up	Baseline	Follow-up			
Primary Outcome							
FHSQ Pain^b^	52.6 (15.7)	61.5 (16.4)	51.1 (16.7)	50.7 (17.6)	9.6 (5.5 to 13.3)	<0.01	0.64
Secondary Outcomes							
Pain VAS (mm)^c^	55.7 (17.9)	45.4 (19.6)	52.7 (18.3)	51.8 (20.5)	−9.4^a^ (5.6 to 13.2)	<0.01	0.33
FHSQ Function^b^	60.7 (20.4)	68.1 (19.4)	60.0 (20.2)	59.1 (21.4)	8.4^a^ (4.8 to 11.7)	<0.01	0.44
FHSQ General Foot Health^b^	26.5 (27.9)	36.7 (30.7)	23.8 (27.0)	25.1 (27.0)	8.9^a^ (0.6 to 13.3)	<0.01	0.41

^a^meets minimal important difference level for a clinically meaningful difference

^b^FHSQ_Foot Health Status Questionnaire (0 = “worst foot health,” 100 = “best foot health”)

^c^VAS_Visual Analog Scale (0 to 100 - higher values indicate greater levels of pain)

### Secondary outcomes

Compared to the control group, participants in the intervention group showed a statistically significant improvement in pain on the VAS at 12 week follow-up with an adjusted mean difference of 9.37 mm and a small effect size (Table 2). They also showed statistically significant improvements in foot function as measured by the FHSQ domain of foot function as well as the domain of general foot health, with effect sizes approaching moderate (Table 2). The between group differences in the scores for all the secondary outcome measures exceeded the MIDs for these scales (Table 2).

Overall comfort of the intervention footwear measured by the VAS scale was rated at 72.1 points (SD 12.3) with a range 43.0 to 95.0. Heel comfort was rated at 67.6 points (SD 13.6) with a range of 42.0 to 93.0. Forefoot comfort was rated at 69.2 points (SD 12.2) with a range of 32.0 to 90.0 and the midfoot comfort was rated a 74.0 points (SD 14.9) with a range of 34.0 to 100.

## Discussion

This trial demonstrated that the use of a flip-flop with a moulded foot-bed resulted in a statistical reduction in pain measured by the FHSQ compared to a group not using a contoured flip-flop. These results are similar to a previous trial by Vincenzio et al. [15] who reported both statistically and clinically important improvements in pain and function following the use of a contoured sandal compared to a flat flip-flop. The reduced effect on pain demonstrated in this present trial compared to that of Vincenzio et al. [15] may have been due to the control condition being the participants’ own footwear rather than a flat flip-flop, a wide inclusion criteria for this trial, a relatively low adherence to the intervention or short trial duration for foot pain that was frequently chronic in nature. However, it should be noted that while there is no normative data for the questionnaires used in both these trials, the post-trial scores are noticeably less than the possible maximum indicating that this footwear intervention is an adjunct rather than a primary treatment for foot pain.

This study also demonstrated a statistically significant improvement in secondary measures of foot function, general foot health and foot pain measured by the VAS. All of these measures also showed clinically significant between group differences, based on validated minimal important differences for these assessment tools [21]. Overall the use of the flip flops demonstrated a positive effect on perceptions of foot pain and function. However, it should be noted that as secondary outcome measures it is possible that this study was underpowered to detect significant differences between groups, increasing the risk of a Type 1 error.

This study did not record any measures of foot and lower limb kinetics and kinematics. However, changes in variables such as plantar pressures and motion control may have contributed to the improvements in pain. High plantar pressures during gait have been linked to the development of foot pain in a variety of populations [5, 29] and the use of traditional flat flip-flops have been shown to produce higher peak plantar pressures than athletic shoes [11]. Conversely, contoured orthoses have been shown to decrease peak pressure under the rearfoot and forefoot by increasing the maximum force and contact area under the midfoot [30, 31]. It is plausible that the moulded foot-bed in the intervention flip-flop may act in the same way to assist in the reduction and redistribution of plantar pressures, and so reduce high forefoot pressure which has been associated with foot pain [31]. While research investigating the change in gait kinematics and kinetics with flip-flop style footwear is limited there is some evidence to suggest that flip-flop style footwear results in changes to walking parameters such as shortened stride length [32] and slower walking speeds [33]. Lower plantar pressures have been documented with slower walking speeds in both younger and older adults [34] as well as with decreased stride length [35], and it is possible that these changes may have contributed to the reduction in reported pain levels. The test flip-flop footwear also featured a small bevel to the anterior and posterior sole of the shoe, effectively making a rocker sole, which has been shown to reduce forefoot plantar pressures and restrict sagittal plane range of motion in painful joints [36]. It has also been reported that many people wear shoes that are too narrow and short for their foot which can contribute to foot pain by increasing the risk of ulceration, callus, corns and toe deformity [37, 38]. The open style of the intervention flip-flops may have added to the reduction in foot pain through better accommodation of the foot and reduction in pressure on bony prominences.

The footwear was well tolerated by the participants in the intervention group with high comfort levels reported for both overall comfort as well as perceived comfort in the separate sub regions of the forefoot, midfoot and rearfoot. Shoe fit is known to influence levels of perceived shoe comfort as tightly fitting shoes can result in uncomfortable foot compression, while loose fitting shoes can lead to soft tissue injury by slippage and friction [39]. The contoured midsole and the open style of the intervention flip-flops may have contributed to a better perceived fit and the high reported comfort levels. In addition, the potential redistribution of plantar pressure with the moulded footwear may also have increased perceived comfort as high forefoot pressure and smaller contact area in the midfoot has been associated with poor footwear comfort ratings [40]. Further, arch comfort has been identified by one study as the most important influence in overall footwear comfort [25] and this area recorded the highest comfort level rating in our study.

### Limitations

The results of this study need to be considered in light of several limitations. It was not possible for participants to be blinded as they were asked to wear either their own shoes or the intervention shoes for 12 weeks. This may have resulted in the participants in the intervention group having a more positive response to the intervention. This may have led to a placebo effect, ascertainment bias or resentful demoralisation which could have impacted the estimate of the treatment effect provided by the intervention [41].

Despite the reported high levels of tolerance and comfort in wearing the moulded foot-bed flip-flops, the adherence to the intervention was relatively low with participants wearing the device for only 15 h per week. This possibly reflects the limitation of the intervention footwear with its open style design which makes it unsafe, or inappropriate in some workplace and social situations and that the weather influences choice of footwear. While this clearly limits the application of this modality it is possible that a larger magnitude in pain reduction may be achieved with a sub group of people whose lifestyle allows more frequent usage of open footwear.

As previously stated, it is possible the full extent of pain reduction was not achieved in the 12 week course of the trial and that longer periods would have created greater changes in foot pain and function. However it is also possible that the reductions in foot pain and improvements in foot function may have diminished with a longer duration as this has been shown to occur with orthotic use for people with plantar fasciitis [42]. The wide range of conditions causing foot pain for participants in this trial may also have affected the results of this study as certain conditions such as arthritis may respond differently to footwear than conditions that tend to be more transient such as plantar fasciitis [14, 43]. As any type of foot pain was included it is also possible that conditions associated with poor fit of enclosed shoes responded well to open style footwear and the reductions in pain were not related to function of the footwear but lack of pressure. Even if this is the case, the reduction in pain is still relevant as poor fitting shoes are a major contributor to foot pain in the community [44]. Further research would help to determine pathology explicit responses to this type of footwear. In addition, the method of data collection required participants to self-report their daily footwear use. While self-report is a simple and inexpensive method of reporting it has been associated with over-estimation in medication and exercise trials [45, 46].

## Conclusions

This study was undertaken to assess the effectiveness of the flip-flop footwear with a moulded foot-bed for reducing foot pain, improving foot function and perception of general foot health and to determine the perceived comfort of test footwear. Our results show that specialised flip-flop footwear had a statistically significant effect on foot pain but the reduction in pain did not meet the minimal important difference for a clinical effect. However, the trial demonstrated global improvements in all aspects of pain and function and perceived foot health indicating that flip flops with a moulded foot bed might be a valuable adjunct therapy for people with foot pain. Features of the test flip-flop such as a moulded foot-bed, bevel and open style may be contributing factors to the reduction in foot pain.

## Abbreviations

ANCOVA: Analysis of covariance
FHSQ: Foot Health Status Questionnaire
MID: Minimal important difference
QOL: Quality of life
SD: Standard deviation
VAS: Visual analog scale
